# Supplementary material for: Physical activity, acute severity and long-term consequences of COVID-19: an 18-month follow-up survey based on a Swedish national cohort
Source: BMJ Open. 2024 Jan 4;14(1):e079927. doi: 10.1136/bmjopen-2023-079927 (PMC10773417; doi:10.1136/bmjopen-2023-079927)
Supplement: Author's manuscript [file bmjopen-2023-079927.draft_revisions.pdf]

BMJ Open is committed to open peer review. As part of this commitment we make the peer review history of every article we publish publicly available.

When an article is published we post the peer reviewers' comments and the authors' responses online. We also post the versions of the paper that were used during peer review. These are the versions that the peer review comments apply to.

The versions of the paper that follow are the versions that were submitted during the peer review process. They are not the versions of record or the final published versions. They should not be cited or distributed as the published version of this manuscript.

BMJ Open is an open access journal and the full, final, typeset and author-corrected version of record of the manuscript is available on our site with no access controls, subscription charges or pay-per-view fees (http://bmjopen.bmj.com).

If you have any questions on BMJ Open's open peer review process please email info.bmjopen@bmj.com

BMJ Open

The Impact of Physical Activity Level prior to COVID-19 on Acute Severity and Long-term Consequences: an 18-month Follow-up Survey of a National Cohort

Journal:	*BMJ Open*
Manuscript ID	bmjopen-2023-079927
Article Type:	Original research
Date Submitted by the Author:	15-Sep-2023

Complete List of Authors:	Palstam, Annie; University of Gothenburg, Department of Clinical Neuroscience; Sahlgrenska University Hospital, Department of Neuroscience<br>Seljelid, Johanna; University of Gothenburg, Department of Clinical Neuroscience<br>Persson, Hanna; University of Gothenburg, Department of Clinical Neuroscience; Sahlgrenska University Hospital, Department of Occupational Therapy and Physical Therapy<br>Sunnerhagen, Katharina; University of Gothenburg, Department of Clinical Neuroscience; Sahlgrenska University Hospital, Department of Neuroscience

Keywords:	COVID-19, Public health < INFECTIOUS DISEASES, Patient Reported Outcome Measures

SCHOLARONE™
Manuscripts

I, the Submitting Author has the right to grant and does grant on behalf of all authors of the Work (as defined in the below author licence), an exclusive licence and/or a non-exclusive licence for contributions from authors who are: i) UK Crown employees; ii) where BMJ has agreed a CC-BY licence shall apply, and/or iii) in accordance with the terms applicable for US Federal Government officers or employees acting as part of their official duties; on a worldwide, perpetual, irrevocable, royalty-free basis to BMJ Publishing Group Ltd ("BMJ") its licensees and where the relevant Journal is co-owned by BMJ to the co-owners of the Journal, to publish the Work in this journal and any other BMJ products and to exploit all rights, as set out in our licence.

The Submitting Author accepts and understands that any supply made under these terms is made by BMJ to the Submitting Author unless you are acting as an employee on behalf of your employer or a postgraduate student of an affiliated institution which is paying any applicable article publishing charge ("APC") for Open Access articles. Where the Submitting Author wishes to make the Work available on an Open Access basis (and intends to pay the relevant APC), the terms of reuse of such Open Access shall be governed by a Creative Commons licence – details of these licences and which Creative Commons licence will apply to this Work are set out in our licence referred to above.

Other than as permitted in any relevant BMJ Author's Self Archiving Policies, I confirm this Work has not been accepted for publication elsewhere, is not being considered for publication elsewhere and does not duplicate material already published. I confirm all authors consent to publication of this Work and authorise the granting of this licence.

For peer review only

The Impact of Physical Activity Level prior to COVID-19 on Acute Severity and Long-term Consequences: an 18-month Follow-up Survey of a National Cohort

Authors: Annie Palstam [1,2,3], Johanna Seljelid [1], Hanna C Persson [1,4], Katharina S Sunnerhagen[1,2]

Affiliations

[1] Department of Clinical Neuroscience, Institute of Neuroscience and Physiology, Sahlgrenska Academy, University of Gothenburg, Sweden; [2] Department of Neuroscience, Sahlgrenska University Hospital, Gothenburg, Sweden; [3] School of Health and Welfare, Dalarna University, Falun, Sweden; [4] Department of Occupational Therapy and Physical Therapy, Sahlgrenska University Hospital, Gothenburg, Sweden

Email address to all

annie.palstam@gu.se; gusseljjo@student.gu.se; hanna.persson@neuro.gu.se; ks.sunnerhagen@neuro.gu.se

Statements relating to ethics and integrity policies

Data availability statement: Complete data cannot be made publicly available for ethical and legal reasons according to the Swedish regulations of the "Act concerning the Ethical Review of Research Involving Humans (2006:460)" and the Swedish Ethical Reviews Authority (http://www.epn.se). Permission to use data is only for what has been given ethical approval by the Swedish Ethical Review Authority. Upon reasonable request, data may be available from the Department of Clinical Neuroscience, SU/Sahlgrenska, Blå stråket 7, plan 3, SE-413 45 Göteborg, Sweden. Email: inf@neuro.gu.se.

Funding statement: This study has been funded by the AFA Insurance [grant number; 200324], and the Swedish state under the agreement between the Swedish government and the county council, the ALF agreement [grant numbers; 965653 and 942914].

Conflict of interest disclosure: Disclosure: none.

Ethics approval statement: This study was approved by the Swedish Ethical Review Authority on June 24, 2020 (2020-03046), with an amendment approved on September 1, 2021 (2021-03556).

Patient consent statement: Informed consent was given by responding to the questionnaire.

Permission to reproduce material from other sources: We have not used material from other sources in this study.

Clinical trial registration: This study is registered at the Researchweb.org, project number 274476.

For peer review only - http://bmjopen.bmj.com/site/about/guidelines.xhtml

For peer review only

For peer review only - http://bmjopen.bmj.com/site/about/guidelines.xhtml

Abstract

Objective

The aim was to investigate how changes in levels of physical activity (PA) relate to acute disease severity and to perceived difficulties in performing daily life activities 18 months after COVID-19 infection.

Design

This is a cross-sectional, observational study.

Participants

The study combined register and survey data on a Swedish national cohort of 11,955 individuals on sick leave due to COVID-19 during the first wave of the pandemic, with an 18-month follow-up. The follow-up survey included questions on daily life activities as well as present and retrospective level of PA.

Outcomes

Changes in PA level were assessed with the Saltin–Grimby PA Level Scale (SGPALS) from before COVID-19 to follow-up and analysed with the Wilcoxon signed-rank test; comparisons of groups were analysed with the Student t-test, Mann–Whitney U test, and chi-square test. Multiple binary logistic regression was performed to assess the value of change in PA in explaining perceived difficulties in performing daily life activities.

Results

In the 5464 survey responders, the PA level was found to decrease. Hospitalised individuals had a lower level of PA both prior to COVID-19 ($p = 0.035$) and at the 18-month follow-up ($p = 0.008$), compared with non-hospitalised responders; however, the level of PA decreased in both groups. A decrease in PA level increased the odds (OR 5.58, 95% CI 4.90–6.34) of having difficulties in performing daily life activities.

Conclusions

PA levels were reduced 18 months after COVID-19 infection. The COVID-19 pandemic has spotlighted the importance of PA in maintaining physical and mental well-being. Since PA is important in maintaining health, and deconditioning takes time to reverse, this decline may have long-term implications for PA and health.

For peer review only - http://bmjopen.bmj.com/site/about/guidelines.xhtml

Strengths and limitations of this study

- A strength of this study was the comprehensive national cohort including all people registered with sick leave during the first pandemic wave in Sweden.
- A limitation to this study was the response rate to the 18-month follow-up survey of nearly fifty percent, where differences between responders and non-responders needs to be considered when interpreting the findings.
- Another strength of this study was the use of a validated instrument for assessing PA level, with clearly defined questions which lends weight to the results and facilitates comparisons with other studies.
- Another limitation to this study was the retrospective assessment of PA level, asking responders to report their PA level 18 months previously, involving risk or recall bias.

For peer review only

Introduction

The COVID-19 pandemic has spotlighted the importance of physical activity (PA) in maintaining physical and mental well-being while enduring lockdowns and periods of social isolation (1, 2). COVID-19 has been found to cause a variety of different acute symptoms (3) of varying severity; the vast majority of patients have a mild infection, while some have a more severe infection requiring acute hospital treatment (4). For some, the symptoms from the acute COVID-19 infection remain, with a multitude of long-term consequences (5, 6). Limitations in usual activities have been reported as the third most prevalent symptom (14.9%) up to 1 year after infection in populations with both hospitalised and non-hospitalised individuals (7). In a small Swedish study of individuals with post–COVID-19 condition, 62% reported having difficulties being physically active 2 years post discharge from hospital, although there was a significant improvement from the 4-month follow-up (8).

To be regularly physically active is a well-known strategy recommended for promoting health and preventing disease (9). Regular PA can also strengthen the immune system, which has been suggested to be beneficial when exposed to viral communicable diseases (10), including COVID-19 (11). When infected with COVID-19, physically inactive individuals have been found to be at higher risk for more severe illness, hospitalisation, and death than are individuals who are regularly physically active (11, 12).

Several studies (13-18) have reported on changes in PA during the pandemic. Although multiple studies with Swedish populations (13-15) have reported at least a slight increase in PA, other studies from the United Kingdom (16), Italy (17), and the USA (18) have reported a decrease. This may be

For peer review only - http://bmjopen.bmj.com/site/about/guidelines.xhtml

partly explained by different approaches to restrictions during the pandemic (14). The changes in PA also seem to differ depending on age(13, 16), sex (13, 17), occupation (13), and the different waves of the pandemic. A Swedish study (13) reported that more individuals changed their lifestyle habits during the first wave than during the second, with 26.4% reporting a negative change in daily activity during the first wave, compared with 20.3% during the second. Furthermore, studies have shown that decreased PA was associated with reduced life satisfaction (14) and a negative impact on mental health(13, 17, 18), and that social inequality in PA has increased during the pandemic (15).

In the working-age population, falling ill with a COVID-19 infection often means having to take sick leave from work (19). For some, this results in long periods of sick leave and sometimes periods of recurrent sick leave (20, 21). In Sweden, compensation for sick leave is comprehensive, tax funded, and administered by the Swedish Social Insurance Agency (SSIA). Our previous results revealed that nearly 12,000 individuals in Sweden were on sick leave due to COVID-19 within the framework of sickness benefits during the first pandemic wave (20). These individuals comprise the cohort in the current study.

Based on this national cohort of individuals registered with sick leave due to COVID-19 during the first pandemic wave, we aimed to investigate how changes in PA levels in regard to acute disease severity relate to perceived difficulties in performing daily life activities 18 months after COVID-19 infection.

Materials and Methods

Study population and setting

This study is a long-term follow-up survey of a national cohort including all individuals in Sweden with sick leave due to COVID-19 (11,955 individuals) during the first wave of the pandemic (20). The inclusion criterion was sick leave due to a COVID-19 diagnosis (International Statistical Classification of Diseases ICD code U07) registered in the national SSIA from March 1 through August 31, 2020 and includes all cases. Registration with the SSIA starts at 2 weeks of sickness absence for individuals who are employed. If self-employed or unemployed, individuals are registered with the SSIA from the start of sickness absence. In this study, receiving sickness benefits regardless of amount was defined as sick leave. The study combines long-term follow-up survey data and data from national registers.

Patient and Public Involvement

For peer review only - http://bmjopen.bmj.com/site/about/guidelines.xhtml

A patient partner were not involved in the present study. However, two patient partners participate within the research group and are involved in research regarding Covid-19.

Data collection

Registry data

The SSIA provided the study population and sick-leave data for the study cohort. The sick-leave period due to COVID-19 was the number of days registered with sickness benefits that included at least one registration with a COVID-19 diagnosis. Sick leave during the year prior to COVID-19 was defined as being registered with sick leave for ≥28 days or ≥6 times between March 1, 2019, and the start of sick leave due to COVID-19. The National Board of Health and Welfare keeps the National Patient Registry including all inpatient care in Sweden. The Registry contributed data on in-hospital care due to COVID-19, which was defined as at least 1 day of hospital stay with registration of any U07 diagnoses. In-hospital care due to COVID-19 was used as a proxy for acute disease severity in the present study. Statistics Sweden provided data on education and income levels, where the individual disposable income during the year prior to COVID-19 (2019) was retrieved, and presented in tertiles of low, medium- and high income.

Survey data

Approximately 18 months after the start of initial sick leave registration due to COVID-19, the follow-up survey was distributed to the cohort. The distribution was conducted by the survey company Indicator (Institutet för kvalitetsindikatorer) with access only to postal addresses and telephone numbers of the cohort. A postal invitation letter to participate in the survey was first distributed on February 25, 2022. The options provided for completing the survey were over the web or by post. This was followed up with two reminders by text messaging and one by postal mail. The survey was open for completion to April 24, 2022. Informed consent was given by responding to the questionnaire. The study was approved by the Swedish Ethical Review Authority on June 24, 2020 (2020-03046), with an amendment approved on September 1, 2021 (2021-03556). Survey data were merged with cohort data by code, and researchers had access only to pseudonymised data. The code key is kept by Statistics Sweden.

The questionnaire included several questions regarding remaining symptoms due to COVID-19, perceived recovery and functioning in daily life, and PA levels before COVID-19 and at follow-up. For the purpose of this study, questions on past and current PA levels as well as an item on long-term consequences of COVID-19 for everyday life were included.

For peer review only - http://bmjopen.bmj.com/site/about/guidelines.xhtml

Current PA level, as well as PA level prior to COVID-19, was reported using the standardised Saltin–Grimby PA Level Scale (SGPALS) (22, 23), which is scored at four levels: 1, physically inactive; 2, some light PA for at least 4 hours/week; 3, regular moderate PA and training for at least 2–3 hours/week; and 4, regular vigorous physical training for competitive sports several times/week. Long-term consequences of COVID-19 were reported through the question "Do you have difficulties in performing daily life activities due to COVID-19?" (yes/no) and/or by grading difficulties on a 3-point scale (small, some, or big difficulties).

Statistics

Data management and analysis were performed in SPSS Statistics 25 (IBM). Descriptive statistics are presented as frequencies and percentages, means with standard deviations (SD), or medians with interquartile range (IQR). Analyses of group comparisons, including drop-out analysis, were conducted using the Student t-test with continuous variables and the Mann–Whitney U test or chi-square test with categorical variables. Analyses of changes in PA within groups were performed using the Wilcoxon signed-rank test. A Sankey diagram was created to graphically illustrate changes in PA. Multiple binary logistic regression was performed to assess the explanatory value of change in PA for perceived difficulties in performing daily life activities, and the results are presented in a forest plot. Variables included in the model were change in PA level from prior COVID-19 infection up until the 18-month follow-up, age, sex, initial hospitalisation, and sick leave during the year prior to COVID-19 infection. The significance level was set to $p < 0.05$.

Results

Study population

Out of 11,955 individuals in the cohort, 5464 individuals completed the survey, corresponding to a response rate of 45.7%, whereof 4240 completed the survey via the web. The mean age of the responders was 51 years, whereof 66% were women. Among responders, the mean number of days on sick leave during the first year following COVID-19 infection was 67, and approximately one in four had been hospitalised due to COVID-19 (Table 1).

Responders were older, had longer duration of sick leave due to COVID-19, were more likely to have been hospitalised due to COVID-19, were more often women, and had more education and income than non-responders (Table 1). There was no significant difference in sick leave prior to COVID-19 between responders and non-responders (Table 1).

For peer review only - http://bmjopen.bmj.com/site/about/guidelines.xhtml

For peer review only

TABLE 1 Characteristics of responders and non-responders.

	Responders *n* = 5464 (45.7%)	Non-responders *n* = 6491 (54.3%)	*p*-value
**Age**, mean (SD)	50.85 (10.21)	45.60 (11.62)	<0.001
**Sex**, *n* (%)			
Men	1865 (34.1)	2961 (45.6)	
Women	3599 (65.9)	3530 (54.4)	<0.001
**Education level**, *n* (%)			
Primary school (≤9 years)	347 (6.4)	890 (13.9)	
Secondary school (10–12 years)	2598 (47.6)	3291 (51.3)	
Short university education (13–14 years)	841 (15.4)	902 (14.1)	<0.001
Long university education (≥15 years)	1667 (30.6)	1328 (20.7)	
**Employment status**, *n* (%)			
Employed	5384 (98.5)	6306 (97.1)	
Unemployed	80 (1.5)	185 (2.9)	<0.001
**Income**, *n* (%)			
Low income	1428 (26.1)	2555 (39.4)	
Medium income	1900 (34.8)	2085 (32.1)	
High income	2136 (39.1)	1849 (28.5)	<0.001
**Hospitalisation due to COVID-19**, *n* (%)	1503 (27.5)	1457 (22.4)	<0.001
**Days of sick leave**			
Mean (SD)	67.28 (91.80)	57.80 (74.55)	<0.001
Median (IQR)	36 (28)	35 (24)	0.003
**Sick leave prior to COVID-19**, *n* (%)	856 (15.7)	1076 (16.6)	0.178

Abbreviations: SD, standard deviation; IQR, interquartile range

Physical activity

PA level was lower in hospitalised responders both prior to COVID-19 ($p = 0.035$) and at the 18-month follow-up ($p = 0.008$) than it was in non-hospitalised responders (Table 2).

TABLE 2. Current and previous physical activity levels in the total group of responders, as well as in subgroups of initially hospitalised and non-hospitalised responders.

	Responders	Responders hospitalised due to COVID-19	Responders non-hospitalised due to COVID-19	*p*-value
**Physical activity level prior to COVID-19 infection, *n* (%)**	5175 (94.7)	1438 (95.6)	3737 (94.3)	
Vigorous physical activity, *n* (%)	455 (8.8)	111 (7.7)	344 (9.2)	
Moderate physical activity, *n* (%)	2085 (40.3)	560 (38.9)	1525 (40.8)	
Light physical activity, *n* (%)	2252 (43.5)	642 (44.6)	1610 (43.1)	0.035
Physically inactive, *n* (%)	383 (7.4)	125 (8.7)	258 (6.9)	
**Current physical activity level, *n* (%)**	5187 (94.9)	1437 (95.6)	3750 (94.7)	
Vigorous physical activity, *n* (%)	159 (3.1)	39 (2.7)	120 (3.2)	0.008

For peer review only - http://bmjopen.bmj.com/site/about/guidelines.xhtml

Moderate physical activity, *n* (%)	1369 (26.4)	395 (27.5)	974 (26.0)	
Light physical activity, *n* (%)	2782 (53.6)	726 (50.5)	2056 (54.8)	
Physically inactive, *n* (%)	877 (16.9)	277 (19.3)	600 (16.0)	

In the whole group of responders, the PA level was found to decrease from prior COVID-19 infection to the 18-month follow-up ($p < 0.001$) (Figure 1). Summing up changes in PA, the level of PA decreased in 1726 (33.6%) responders, was sustained in 3105 (60.5%), and increased in 300 (5.8%). Of the individuals with the highest level of PA (SGPALS 4) prior to COVID-19, approximately one-third sustained their level of PA until the 18-month follow-up. In subgroup analysis based on acute disease severity, PA level was found to decrease in the non-hospitalised group ($p < 0.001$), where 1244 responders (33.5%) decreased their level of PA, 2282 (61.5%) sustained their PA level, and 184 (5.1%) increased their PA level. A significant decrease in PA level was also found in the initially hospitalised responders ($p < 0.001$), where 482 (33.9%) decreased their level of PA, 823 (58.0%) sustained their PA level, and 115 (8.1%) increased their level of PA.

Insert figure 1 about here

Long-term consequences of COVID-19 in daily life and their relation to PA

At the 18-month follow-up, 2434 responders (46%) reported still having difficulties in performing daily life activities due to COVID-19. Logistic regression was performed to explain these long-term difficulties in performing daily life activities due to COVID-19. The final model showed that a decrease in PA level increased the odds (OR 5.58, 95% CI 4.90–6.34) of having difficulties in performing daily life activities. Furthermore, having been initially hospitalised due to COVID-19 and having had sick leave during the year prior to COVID-19 also increased the odds, while age and sex did not contribute significantly to long-term difficulties in daily life activities (Figure 2). The final model could significantly ($p < 0.001$) distinguish between responders who did or did not report perceived difficulties in performing daily life activities at the 18-month follow-up.

Insert figure 2 about here

Discussion

The results of this study show that individuals with less severe acute disease had a higher level of PA, both prior to COVID-19 and at the 18-month follow-up, than did individuals with more severe acute disease. In both the total study population and in both groups based on acute disease severity, the

For peer review only - http://bmjopen.bmj.com/site/about/guidelines.xhtml

PA level was found to decrease from prior to COVID-19 to the 18-month follow-up. Furthermore, a decrease in PA level over that time was found to explain perceiving difficulties with performing daily life activities at 18 months after COVID-19.

Among responders, the PA level before COVID-19 was rather high. The study population also seems to be quite physically active in comparison with other cohorts in which PA was assessed by the same measure (24-26). Given that regular PA has been shown to be beneficial in protecting individuals who fall ill with COVID-19 against severe illness and hospitalisation (11, 12), one might anticipate that this population would not be severely affected by COVID-19 infection. Nevertheless, one-fourth of the current study population were initially hospitalised. Being more physically active during the pandemic may have entailed being in places or contexts with a higher risk of transmission of COVID-19, such as gyms or practice in team sports, while less active individuals may have stayed more isolated in their own homes. Furthermore, exercising while already infected with COVID-19 may have worsened the course of the disease (27). From the polio epidemics, being very physically active (28) or pregnant (29) (both affect the immune system) increased the risk of paralysis among poliomyelitis cases. However, in the comparison the current study population's subgroups based on acute disease severity, hospitalised responders had significantly lower PA than did non-hospitalised, supporting previous findings of the benefits of PA for COVID-19 severity (11, 12).

Summing up changes in PA, there was a decrease both in the total study population and in the groups based on acute disease severity. In the general Swedish population, PA has been found to have increased during the pandemic(13-15), in contrast to the current findings in a cohort of individuals who have had COVID-19. It can be speculated that the decrease in PA seen in the current study is related to the disease itself and the deconditioning thereof (30). However, a Swedish study (14) reported that in a general population, 36% reported increased and 30% decreased PA during the pandemic. Of these, 71% and 89%, respectively, attributed this change to COVID-19–related restrictions (14). The relatively mild approach to restrictions in Sweden may have had diverse effects on PA in different groups of individuals; recommendations, such as working from home, may have given more free time and therefore increased PA in some, while cancelled sporting events and competitions may have had the opposite effect, especially among individuals with a high level of PA. In the current study, only one-third of individuals with the highest level of PA prior to COVID-19 sustained their PA level until the 18-month follow-up.

Furthermore, the strictest recommendations, involving isolation and applied to elderly and those infected as a means to protect the most vulnerable and constrain transmission, have most likely had an influence on levels of PA. A study from the US showed an association between self-isolation and

For peer review only - http://bmjopen.bmj.com/site/about/guidelines.xhtml

negative mental health not seen for social distancing (18). Previous studies (13, 17, 18) have also shown an association between negative mental health effects and a decrease in PA during the pandemic. Together, this suggests that pandemic-related restrictions and mental health could also play a role in the decrease in PA found in the current cohort.

A strength of the present study was that a validated instrument, SGPALS, was used for questions regarding PA level. Furthermore, the different steps of the scale were clearly defined in the questionnaire, making it easier for individuals to answer according to their true activity levels. This lends weight to the results and facilitates comparisons with other studies and populations.

In this study, in-hospital care due to COVID-19 was used as a proxy for acute disease severity. As no one in Sweden was denied in-hospital care due to lack of capacity, and healthcare in Sweden is tax-funded, this proxy for acute disease severity seems reasonable and relies on the assessment of educated healthcare professionals. Furthermore, need of hospital care is included in the WHO Clinical Progression Scale (31), which supports the interpretation of in-hospital care as a proxy for COVID-19 disease severity.

Using SGPALS (23), the current study showed a decrease in PA in individuals who were on sick leave due to COVID-19 during the first wave of the pandemic in Sweden. Notably, only one-third of individuals with the highest level of PA prior to COVID-19 sustained their level until the 18-month follow-up. Since making up for deconditioning takes time (32), this decline in PA may have long-term implications, and returning to pre–COVID-19 activities and levels may also be difficult for the most highly trained individuals, such as elite athletes (33).

PA is important in maintaining health and can protect against both non-communicable chronic diseases (34) and communicable diseases (35) such as COVID-19. Therefore, the results of this study, with a decline in PA level regardless of acute disease severity, are worrisome, especially considering the risk of future pandemics.

In conclusion, PA levels were reduced 18 months after COVID-19 infection. The COVID-19 pandemic has spotlighted the importance of PA in maintaining physical and mental well-being. Since PA is important in maintaining health, and deconditioning takes time to reverse, this decline may have long-term implications for PA and health.

Author contribution

AP contributed in conception and design of the study, managed data acquisition and analysis, contributed in interpretation of data and drafted the work.

For peer review only - http://bmjopen.bmj.com/site/about/guidelines.xhtml

JS contributed in interpretation and presentation of data, and drafting the work.
HP contributed in conception, design and acquisition of the study as well as in data management, and interpretation.
KSS contributed in conception, design and acquisition of the study, contributed in interpretation of data.
All authors reviewed the work critically for important intellectual content and approved the final version to be published.

For peer review only

For peer review only - http://bmjopen.bmj.com/site/about/guidelines.xhtml

Figure legends:

FIGURE 1 Changes in levels of physical activity according to the Saltin–Grimby PA Level Scale (SGPALS 1–4) from prior to COVID-19 infection to the 18-month follow-up, including *n* = 5131 individuals responding to both prior and current physical activity levels.
SGPALS Level 1–4: 1, physically inactive; 2, some light PA for at least 4 hours/week; 3, regular moderate PA and training for at least 2–3 hours/week; and 4, regular vigorous physical training for competitive sports several times per week.

FIGURE 2. Variables in the final model explaining odds for perceiving difficulties in performing daily life activities. The model explained between 15% (Cox & Snell R^2) and 20% (Nagelkerke R^2) of the variance in the outcome.

For peer review only

For peer review only - http://bmjopen.bmj.com/site/about/guidelines.xhtml

References

1. Ai X, Yang J, Lin Z, et al. Mental Health and the Role of Physical Activity During the COVID-19 Pandemic. Front Psychol. 2021;12:759987.10.3389/fpsyg.2021.759987.
2. Ravalli S, Musumeci G. Coronavirus Outbreak in Italy: Physiological Benefits of Home-Based Exercise During Pandemic. J Funct Morphol Kinesiol. 2020;5(2).10.3390/jfmk5020031.
3. Gupta A, Madhavan MV, Sehgal K, et al. Extrapulmonary manifestations of COVID-19. Nat Med. 2020;26(7):1017-32.10.1038/s41591-020-0968-3.
4. Wiersinga WJ, Rhodes A, Cheng AC, et al. Pathophysiology, Transmission, Diagnosis, and Treatment of Coronavirus Disease 2019 (COVID-19): A Review. Jama. 2020;324(8):782-93.10.1001/jama.2020.12839.
5. Higgins V, Sohaei D, Diamandis EP, et al. COVID-19: from an acute to chronic disease? Potential long-term health consequences. Crit Rev Clin Lab Sci. 2021;58(5):297-310.10.1080/10408363.2020.1860895.
6. Ballering AV, van Zon SKR, Olde Hartman TC, et al. Persistence of somatic symptoms after COVID-19 in the Netherlands: an observational cohort study. Lancet. 2022;400(10350):452-61.10.1016/s0140-6736(22)01214-4.
7. O'Mahoney LL, Routen A, Gillies C, et al. The prevalence and long-term health effects of Long Covid among hospitalised and non-hospitalised populations: A systematic review and meta-analysis. EClinicalMedicine. 2023;55:101762.10.1016/j.eclinm.2022.101762.
8. Wahlgren C, Forsberg G, Divanoglou A, et al. Two-year follow-up of patients with post-COVID-19 condition in Sweden: a prospective cohort study. Lancet Reg Health Eur. 2023;28:100595.10.1016/j.lanepe.2023.100595.
9. World Health Organization. WHO guidelines on physical activity and sedentary behaviour 2020.
10. da Silveira MP, da Silva Fagundes KK, Bizuti MR, et al. Physical exercise as a tool to help the immune system against COVID-19: an integrative review of the current literature. Clin Exp Med. 2021;21(1):15-28.10.1007/s10238-020-00650-3.
11. Ezzatvar Y, Ramírez-Vélez R, Izquierdo M, et al. Physical activity and risk of infection, severity and mortality of COVID-19: a systematic review and non-linear dose-response meta-analysis of data from 1 853 610 adults. Br J Sports Med. 2022.10.1136/bjsports-2022-105733.
12. Sallis R, Young DR, Tartof SY, et al. Physical inactivity is associated with a higher risk for severe COVID-19 outcomes: a study in 48 440 adult patients. Br J Sports Med. 2021;55(19):1099-105.10.1136/bjsports-2021-104080.
13. Blom V, Lönn A, Ekblom B, et al. Lifestyle Habits and Mental Health in Light of the Two COVID-19 Pandemic Waves in Sweden, 2020. Int J Environ Res Public Health. 2021;18(6).10.3390/ijerph18063313.
14. Eek F, Larsson C, Wisén A, et al. Self-Perceived Changes in Physical Activity and the Relation to Life Satisfaction and Rated Physical Capacity in Swedish Adults during the COVID-19 Pandemic-A Cross Sectional Study. Int J Environ Res Public Health. 2021;18(2).10.3390/ijerph18020671.
15. Brattlöf F, Gustafsson PE, San Sebastián M. Prevalence and change in social inequalities in physical activity before and during the COVID-19 pandemic in Sweden. Int J Equity Health. 2023;22(1):21.10.1186/s12939-023-01835-4.
16. McCarthy H, Potts HWW, Fisher A. Physical Activity Behavior Before, During, and After COVID-19 Restrictions: Longitudinal Smartphone-Tracking Study of Adults in the United Kingdom. J Med Internet Res. 2021;23(2):e23701.10.2196/23701.
17. Maugeri G, Castrogiovanni P, Battaglia G, et al. The impact of physical activity on psychological health during Covid-19 pandemic in Italy. Heliyon. 2020;6(6).
18. Meyer J, McDowell C, Lansing J, et al. Changes in Physical Activity and Sedentary Behavior in Response to COVID-19 and Their Associations with Mental Health in 3052 US Adults. Int J Environ Res Public Health. 2020;17(18).10.3390/ijerph17186469.

For peer review only - http://bmjopen.bmj.com/site/about/guidelines.xhtml

19. Calvo-Bonacho E, Catalina-Romero C, Fernández-Labandera C, et al. COVID-19 and Sick Leave: An Analysis of the Ibermutua Cohort of Over 1,651,305 Spanish Workers in the First Trimester of 2020. Front Public Health. 2020;8:580546.10.3389/fpubh.2020.580546.
20. Westerlind E, Palstam A, Sunnerhagen KS, et al. Patterns and predictors of sick leave after Covid-19 and long Covid in a national Swedish cohort. BMC Public Health. 2021;21(1):1023.10.1186/s12889-021-11013-2.
21. Palstam A, Westerlind E, Sunnerhagen KS, et al. Recurrent sick leave after COVID-19: investigating the first wave of the pandemic in a comprehensive Swedish registry-based study. BMC Public Health. 2021;21(1):1914.10.1186/s12889-021-11918-y.
22. Saltin B, Grimby G. Physiological analysis of middle-aged and old former athletes: comparison with still active athletes of the same ages. Circulation. 1968;38(6):1104-15.
23. Grimby G, Börjesson M, Jonsdottir I, et al. The "Saltin–Grimby physical activity level scale" and its application to health research. Scand J Med Sci Sports. 2015;25:119-25.
24. Mikkilä S, Johansson J, Nordström A, et al. A 15-year follow-up study of hip bone mineral density and associations with leisure time physical activity. The Tromsø Study 2001-2016. PLoS One. 2022;17(1):e0262228.10.1371/journal.pone.0262228.
25. Rödjer L, I HJ, Börjesson M. Physical activity on prescription (PAP): self-reported physical activity and quality of life in a Swedish primary care population, 2-year follow-up. Scand J Prim Health Care. 2016;34(4):443-52.10.1080/02813432.2016.1253820.
26. Gustavsson J, Mehlig K, Leander K, et al. FTO genotype, physical activity, and coronary heart disease risk in Swedish men and women. Circ Cardiovasc Genet. 2014;7(2):171-7.10.1161/circgenetics.111.000007.
27. Nieman DC. Coronavirus disease-2019: A tocsin to our aging, unfit, corpulent, and immunodeficient society. Journal of sport and health science. 2020;9(4):293-301.
28. Friman G, Wesslén L. Infections and exercise in high-performance athletes. Immunology and cell biology. 2000;78(5):510-22.
29. Siegel M, Greenberg M. Incidence of poliomyelitis in pregnancy: its relation to maternal age, parity and gestational period. New England Journal of Medicine. 1955;253(20):841-7.
30. Rinaldo RF, Mondoni M, Parazzini EM, et al. Deconditioning as main mechanism of impaired exercise response in COVID-19 survivors. Eur Respir J. 2021;58(2).10.1183/13993003.00870-2021.
31. WHO Working Group on the Clinical Characterisation and Management of COVID-19 infection. A minimal common outcome measure set for COVID-19 clinical research. Lancet Infect Dis. 2020;20(8):e192-e7.10.1016/s1473-3099(20)30483-7.
32. Blomquist G. Respons to exercise after bed rest and training. Circulation. 1968;38(7).
33. Williams Z, Hull JH. Respiratory complications following COVID-19 in athletic populations: A narrative review. Scandinavian Journal of Medicine & Science in Sports. 2022.
34. Lee IM, Shiroma EJ, Lobelo F, et al. Effect of physical inactivity on major non-communicable diseases worldwide: an analysis of burden of disease and life expectancy. Lancet. 2012;380(9838):219-29.10.1016/s0140-6736(12)61031-9.
35. Chastin SFM, Abaraogu U, Bourgois JG, et al. Effects of Regular Physical Activity on the Immune System, Vaccination and Risk of Community-Acquired Infectious Disease in the General Population: Systematic Review and Meta-Analysis. Sports Med. 2021;51(8):1673-86.10.1007/s40279-021-01466-1.

For peer review only - http://bmjopen.bmj.com/site/about/guidelines.xhtml

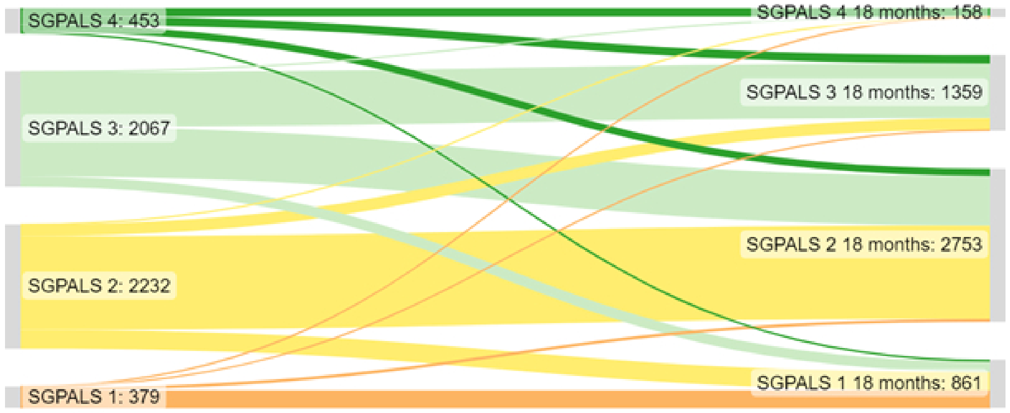

FIGURE 1 Changes in physical activity (PA) levels according to the Saltin–Grimby PA Level Scale (SGPALS) from prior to COVID-19 infection (left side) to the 18-month follow-up (right side) n = 5131 individuals. SGPALS: 1, physically inactive; 2, light PA for at least 4 hours/week; 3, regular moderate PA and training for at least 2–3 hours/week; and 4, regular vigorous physical training for competitive sports several times per week.

404x187mm (38 x 38 DPI)

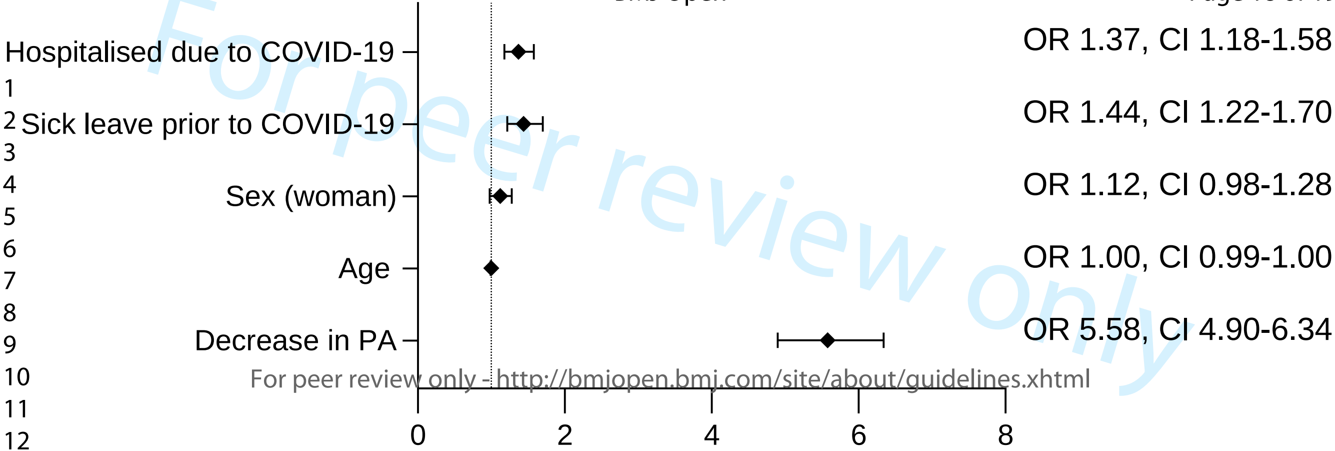

For peer review only
For peer review only - http://bmjopen.bmj.com/site/about/guidelines.xhtml

STROBE 2007 (v4) checklist of items to be included in reports of observational studies in epidemiology*
Checklist for cohort, case-control, and cross-sectional studies (combined)

Section/Topic	Item #	Recommendation	Reported on page #
**Title and abstract**	1	(*a*) Indicate the study's design with a commonly used term in the title or the abstract	1
		(*b*) Provide in the abstract an informative and balanced summary of what was done and what was found	2
**Introduction**			
Background/rationale	2	Explain the scientific background and rationale for the investigation being reported	3-4
Objectives	3	State specific objectives, including any pre-specified hypotheses	4
**Methods**			
Study design	4	Present key elements of study design early in the paper	4
Setting	5	Describe the setting, locations, and relevant dates, including periods of recruitment, exposure, follow-up, and data collection	4-5
Participants	6	(*a*) *Cohort study*—Give the eligibility criteria, and the sources and methods of selection of participants. Describe methods of follow-up<br>*Case-control study*—Give the eligibility criteria, and the sources and methods of case ascertainment and control selection. Give the rationale for the choice of cases and controls<br>*Cross-sectional study*—Give the eligibility criteria, and the sources and methods of selection of participants	4-5
		(*b*) *Cohort study*—For matched studies, give matching criteria and number of exposed and unexposed<br>*Case-control study*—For matched studies, give matching criteria and the number of controls per case	
Variables	7	Clearly define all outcomes, exposures, predictors, potential confounders, and effect modifiers. Give diagnostic criteria, if applicable	4-5
Data sources/ measurement	8*	For each variable of interest, give sources of data and details of methods of assessment (measurement). Describe comparability of assessment methods if there is more than one group	4-5
Bias	9	Describe any efforts to address potential sources of bias	6
Study size	10	Explain how the study size was arrived at	5
Quantitative variables	11	Explain how quantitative variables were handled in the analyses. If applicable, describe which groupings were chosen and why	6
Statistical methods	12	(*a*) Describe all statistical methods, including those used to control for confounding	6
		(*b*) Describe any methods used to examine subgroups and interactions	6
		(*c*) Explain how missing data were addressed	6
		(*d*) *Cohort study*—If applicable, explain how loss to follow-up was addressed<br>*Case-control study*—If applicable, explain how matching of cases and controls was addressed	6

For peer review only

		Cross-sectional study—If applicable, describe analytical methods taking account of sampling strategy	
		(*e*) Describe any sensitivity analyses	
**Results**			
Participants	13*	(a) Report numbers of individuals at each stage of study—eg numbers potentially eligible, examined for eligibility, confirmed eligible, included in the study, completing follow-up, and analysed	6
		(b) Give reasons for non-participation at each stage	6
		(c) Consider use of a flow diagram	
Descriptive data	14*	(a) Give characteristics of study participants (eg demographic, clinical, social) and information on exposures and potential confounders	6
		(b) Indicate number of participants with missing data for each variable of interest	6-8
		(c) *Cohort study*—Summarise follow-up time (eg, average and total amount)	6
Outcome data	15*	*Cohort study*—Report numbers of outcome events or summary measures over time	
		Case-control study—Report numbers in each exposure category, or summary measures of exposure	
		Cross-sectional study—Report numbers of outcome events or summary measures	6-8
Main results	16	(*a*) Give unadjusted estimates and, if applicable, confounder-adjusted estimates and their precision (eg, 95% confidence interval). Make clear which confounders were adjusted for and why they were included	8
		(*b*) Report category boundaries when continuous variables were categorized	
		(*c*) If relevant, consider translating estimates of relative risk into absolute risk for a meaningful time period	
Other analyses	17	Report other analyses done—eg analyses of subgroups and interactions, and sensitivity analyses	
**Discussion**			
Key results	18	Summarise key results with reference to study objectives	8
Limitations	19	Discuss limitations of the study, taking into account sources of potential bias or imprecision. Discuss both direction and magnitude of any potential bias	3, 9
Interpretation	20	Give a cautious overall interpretation of results considering objectives, limitations, multiplicity of analyses, results from similar studies, and other relevant evidence	10
Generalisability	21	Discuss the generalisability (external validity) of the study results	3, 9-10
**Other information**			
Funding	22	Give the source of funding and the role of the funders for the present study and, if applicable, for the original study on which the present article is based	1

*Give information separately for cases and controls in case-control studies and, if applicable, for exposed and unexposed groups in cohort and cross-sectional studies.

Note: An Explanation and Elaboration article discusses each checklist item and gives methodological background and published examples of transparent reporting. The STROBE checklist is best used in conjunction with this article (freely available on the Web sites of PLoS Medicine at http://www.plosmedicine.org/, Annals of Internal Medicine at http://www.annals.org/, and Epidemiology at http://www.epidem.com/). Information on the STROBE Initiative is available at www.strobe-statement.org.

For peer review only

BMJ Open

Physical activity, acute severity, and long-term consequences of COVID-19: an 18-month follow-up survey based on a Swedish national cohort

Journal:	*BMJ Open*
Manuscript ID	bmjopen-2023-079927.R1
Article Type:	Original research
Date Submitted by the Author:	15-Nov-2023
Complete List of Authors:	Palstam, Annie; University of Gothenburg, Department of Clinical Neuroscience; Sahlgrenska University Hospital, Department of Neuroscience<br>Seljelid, Johanna; University of Gothenburg, Department of Clinical Neuroscience<br>Persson, Hanna; University of Gothenburg, Department of Clinical Neuroscience; Sahlgrenska University Hospital, Department of Occupational Therapy and Physical Therapy<br>Sunnerhagen, Katharina; University of Gothenburg, Department of Clinical Neuroscience; Sahlgrenska University Hospital, Department of Neuroscience
**Primary Subject Heading**:	Public health
Secondary Subject Heading:	Epidemiology, Public health, Rehabilitation medicine
Keywords:	COVID-19, Public health < INFECTIOUS DISEASES, Patient Reported Outcome Measures

SCHOLARONE™
Manuscripts

I, the Submitting Author has the right to grant and does grant on behalf of all authors of the Work (as defined in the below author licence), an exclusive licence and/or a non-exclusive licence for contributions from authors who are: i) UK Crown employees; ii) where BMJ has agreed a CC-BY licence shall apply, and/or iii) in accordance with the terms applicable for US Federal Government officers or employees acting as part of their official duties; on a worldwide, perpetual, irrevocable, royalty-free basis to BMJ Publishing Group Ltd ("BMJ") its licensees and where the relevant Journal is co-owned by BMJ to the co-owners of the Journal, to publish the Work in this journal and any other BMJ products and to exploit all rights, as set out in our licence.

The Submitting Author accepts and understands that any supply made under these terms is made by BMJ to the Submitting Author unless you are acting as an employee on behalf of your employer or a postgraduate student of an affiliated institution which is paying any applicable article publishing charge ("APC") for Open Access articles. Where the Submitting Author wishes to make the Work available on an Open Access basis (and intends to pay the relevant APC), the terms of reuse of such Open Access shall be governed by a Creative Commons licence – details of these licences and which Creative Commons licence will apply to this Work are set out in our licence referred to above.

Other than as permitted in any relevant BMJ Author's Self Archiving Policies, I confirm this Work has not been accepted for publication elsewhere, is not being considered for publication elsewhere and does not duplicate material already published. I confirm all authors consent to publication of this Work and authorise the granting of this licence.

For peer review only

Physical activity, acute severity, and long-term consequences of COVID-19: an 18-month follow-up survey based on a Swedish national cohort

Authors: Annie Palstam [1,2,3], Johanna Seljelid [1], Hanna C Persson [1,4], Katharina S Sunnerhagen[1,2]

Affiliations

[1] Department of Clinical Neuroscience, Institute of Neuroscience and Physiology, Sahlgrenska Academy, University of Gothenburg, Sweden; [2] Department of Neuroscience, Sahlgrenska University Hospital, Gothenburg, Sweden; [3] School of Health and Welfare, Dalarna University, Falun, Sweden; [4] Department of Occupational Therapy and Physical Therapy, Sahlgrenska University Hospital, Gothenburg, Sweden

Email addresses for all

annie.palstam@gu.se; gusseljjo@student.gu.se; hanna.persson@neuro.gu.se; ks.sunnerhagen@neuro.gu.se

Statements relating to ethics and integrity policies

Data availability statement: Complete data cannot be made publicly available for ethical and legal reasons according to Swedish regulations in the “Act concerning the Ethical Review of Research Involving Humans (2006:460)” and the Swedish Ethical Reviews Authority (http://www.epn.se). Permission to use data is only for what has been given ethical approval by the Swedish Ethical Review Authority. Upon reasonable request, data may be available from the Department of Clinical Neuroscience, SU/Sahlgrenska, Blå stråket 7, plan 3, SE-413 45 Göteborg, Sweden. Email: inf@neuro.gu.se.

Funding statement: This study has been funded by the AFA Insurance [grant number 200324] and the Swedish state under the agreement between the Swedish government and county council, the ALF agreement [grant numbers 965653 and 942914].

Conflict of interest disclosure: Disclosure: none.

Ethics approval statement: This study was approved by the Swedish Ethical Review Authority on June 24, 2020 (2020-03046), with an amendment for this study approved on September 1, 2021 (2021-03556). Participants were fully informed about the study and aware that they consented to participation by completing the questionnaire.

Patient consent statement: Informed consent was given by responding to the questionnaire.

For peer review only - http://bmjopen.bmj.com/site/about/guidelines.xhtml

Permission to reproduce material from other sources: We have not used material from other sources in this study.

Clinical trial registration: This study is registered at Researchweb.org, project number 274476, https://www.researchweb.org/is/sverige/project/274476.

For peer review only

For peer review only - http://bmjopen.bmj.com/site/about/guidelines.xhtml

Abstract

Objective

To investigate how changes in levels of physical activity (PA) in regard to acute disease severity relate to perceived difficulties in performing daily life activities 18 months after COVID-19 infection.

Design

An observational study with an 18-month follow-up survey based on registry data from a national cohort.

Participants

5464 responders to the 18-month follow-up survey of a Swedish national cohort of 11,955 individuals on sick leave due to COVID-19 during the first wave of the pandemic.

Outcomes

The follow-up survey included questions on daily life activities, as well as present and retrospective level of PA. Changes in PA level from before COVID-19 to follow-up were assessed by the Saltin–Grimby PA Level Scale (SGPALS) and analysed by the Wilcoxon signed-rank test. Comparisons of groups were analysed by the Student t-test, Mann–Whitney U test, and chi-squared. Multiple binary logistic regression was performed to assess the association of changes in PA with perceived difficulties in performing daily life activities.

Results

Among the 5464 responders (45% of national cohort), the PA level decreased. Hospitalised individuals had a lower PA level both prior to COVID-19 ($p = 0.035$) and at the 18-month follow-up ($p = 0.008$) compared with non-hospitalised responders. However, the level of PA decreased in both groups. A decrease in PA level increased the odds (OR 5.58, 95% CI 4.90–6.34) of having difficulties performing daily life activities.

Conclusions

PA levels were reduced 18 months after COVID-19 infection. A decrease in PA over that time was associated with perceived difficulties performing daily life activities 18 months after COVID-19. As PA is important in maintaining health and deconditioning takes time to reverse, this decline may have long-term implications for PA and health.

For peer review only - http://bmjopen.bmj.com/site/about/guidelines.xhtml

Strengths and limitations of this study

- A strength of this study was the comprehensive national cohort including all people registered for sick leave during the first pandemic wave in Sweden.
- A limitation of this study was the less than 50% response rate to the 18-month follow-up survey, and differences between responders and non-responders need to be considered when interpreting the findings.
- Another strength of this study was the use of a validated instrument to assess the PA level, with clearly defined questions that lend weight to the results and facilitate comparisons with other studies.
- Another limitation was the retrospective assessment of PA level, asking responders to report their PA level 18 months previous, which involves risk or recall bias.

For peer review only

INTRODUCTION

The COVID-19 pandemic has highlighted the importance of physical activity (PA) in maintaining physical and mental well-being while enduring lockdowns and periods of social isolation (1, 2). COVID-19 has been found to cause different acute symptoms (3) of varying severity; the vast majority of patients have a mild infection, but some have a more severe infection requiring acute hospitalisation (4). For some, the symptoms from the acute COVID-19 infection remain, with a multitude of long-term consequences (5, 6). Limitations in usual activities have been reported as the third most prevalent symptom (14.9%) up to 1 year after infection in populations with both hospitalised and non-hospitalised individuals (7). In a small Swedish study of individuals with a post–COVID-19 condition, 62% reported having difficulties being physically active 2 years post-discharge from hospital, though there was a significant improvement from the 4-month follow-up (8).

Being regularly physically active is a well-known strategy recommended to promote health and prevent disease (9). Regular PA can also strengthen the immune system, which has been suggested to be beneficial when exposed to viral communicable diseases (10), including COVID-19 (11). When infected with COVID-19, physically inactive individuals are at higher risk of more severe illness, hospitalisation, and death than individuals who are regularly physically active (11, 12).

Several studies (13-18) have reported changes in PA during the pandemic. Although multiple studies in Swedish populations (13-15) have reported at least a slight increase in PA, other studies from the United Kingdom (16), Italy (17), and USA (18) have reported a decrease. This may be partly explained by different approaches to restrictions during the pandemic (14). The changes in PA also seem to

For peer review only - http://bmjopen.bmj.com/site/about/guidelines.xhtml

depend on age (13, 16), sex (13, 17), occupation (13), and the different waves of the pandemic. A Swedish study (13) reported that more individuals changed their lifestyle habits during the first wave than during the second wave, with 26.4% reporting a negative change in daily activity during the first wave, compared with 20.3% during the second. Furthermore, studies have shown that decreased PA is associated with reduced life satisfaction (14) and a negative impact on mental health (13, 17, 18), and that social inequality in PA increased during the pandemic (15).

In the working-age population, falling ill with COVID-19 often means having to take sick leave from work (19). For some, this results in long periods of sick leave and, sometimes, periods of recurrent sick leave (20, 21). In Sweden, compensation for sick leave is comprehensive, tax funded, and administered by the Swedish Social Insurance Agency (SSIA). Our previous study found that nearly 12,000 individuals in Sweden were on sick leave due to COVID-19 within the framework of sickness benefits during the first pandemic wave (20). These individuals comprise the cohort of the current study.

Based on the national cohort of individuals registered for sick leave due to COVID-19 during the first pandemic wave, we investigated how PA and changes in PA levels in regard to acute disease severity relate to perceived difficulties in performing daily life activities 18 months after a COVID-19 infection.

MATERIALS AND METHODS

Study population and setting

This study is a long-term follow-up survey of a national cohort that comprised all individuals in Sweden who took sick leave due to COVID-19 (11,955 individuals) during the first wave of the pandemic (20). The inclusion criterion was sick leave due to a COVID-19 diagnosis (International Statistical Classification of Diseases ICD code U07) registered in the national SSIA from March 1 through August 31, 2020. Registration with the SSIA starts at 2 weeks of absence due to sickness for individuals who are employed. If self-employed or unemployed, individuals are registered with the SSIA from the start of the absence. In this study, receiving sickness benefits was defined as sick leave regardless of the amount. The study combines long-term follow-up survey data and data from national registers.

Patient and public involvement

A patient partner was not involved in the present study.

For peer review only - http://bmjopen.bmj.com/site/about/guidelines.xhtml

Data collection

The study was approved by the Swedish Ethical Review Authority on June 24, 2020 (2020-03046), with an amendment approved on September 1, 2021 (2021-03556).

Registry data

The SSIA provided the study population and sick-leave data for the study cohort. The sick-leave period due to COVID-19 was the number of days registered for the sickness benefits that included at least one registration with a COVID-19 diagnosis. Sick leave during the year prior to COVID-19 was defined as being registered for sick leave ≥28 days or ≥6 times between March 1, 2019, and the start of sick leave due to COVID-19. The National Board of Health and Welfare maintains the National Patient Registry of all inpatient care in Sweden. The Registry contributed data on in-hospital care due to COVID-19, which was defined as at least 1 day of hospital stay with registration of any U07 diagnoses. In the present study, in-hospital care due to COVID-19 was used as a proxy for acute disease severity; having been hospitalised defined more severe acute disease than not having been hospitalised. Statistics Sweden provided data on education and income levels; the individual disposable income during the year prior to COVID-19 (2019) was retrieved and presented in tertiles of low, medium, and high income.

Survey data

Approximately 18 months after the start of initial sick leave registration due to COVID-19, the follow-up survey was distributed to the cohort by the survey company Indicator (Institutet för kvalitetsindikatorer). The company only had access to postal addresses and telephone numbers for the cohort. A postal invitation letter to participate in the survey was first distributed on February 25, 2022. The options provided for completing the survey were over the web or by post. This was followed up with two reminders by text messaging and one by postal mail. The survey was open for completion until April 24, 2022. Informed consent was given by responding to the questionnaire. Survey data were merged with cohort data by code, and researchers only had access to pseudonymised data. The code key is kept by Statistics Sweden.

The questionnaire included several questions regarding remaining symptoms due to COVID-19, perceived recovery and functioning in daily life, and PA levels before COVID-19 and at follow-up. For the purpose of this study, questions on past and current PA levels, as well as an item on long-term consequences of COVID-19 on everyday life, were included.

For peer review only - http://bmjopen.bmj.com/site/about/guidelines.xhtml

Current PA level and PA level prior to COVID-19 were reported using the standardised Saltin–Grimby PA Level Scale (SGPALS) (22, 23), which is scored at four levels: 1, physically inactive; 2, light PA for at least 4 hours/week; 3, regular moderate PA and training for at least 2–3 hours/week; and 4, regular vigorous physical training for competitive sports several times each week. The long-term consequences of COVID-19 were reported using the question, "Do you have difficulties performing daily life activities due to COVID-19?" which was answered yes/no and/or by grading difficulties on a 3-point scale (small, some, or big difficulties). The variable was dichotomised into having difficulties in daily life activities and not having difficulties in daily life activities.

Statistical analysis

Data management and analyses were performed in SPSS Statistics 25 (IBM). Descriptive statistics are presented as frequencies and percentages, means with standard deviations (SDs), or medians with interquartile range (IQR). Analyses of group comparisons, including a drop-out analysis, were conducted using the Student t-test with continuous variables, the Mann–Whitney U test with ordinal variables, or chi-squared test with categorical variables. Analyses of changes in PA within groups were performed using the Wilcoxon signed-rank test. A Sankey diagram was created to graphically illustrate changes in PA. Multiple binary logistic regression was performed to assess the association of changes in PA with perceived difficulties performing daily life activities, and the results are presented in a forest plot. Variables included in the model were change in PA level from prior COVID-19 infection up until the 18-month follow-up, age, sex, initial hospitalisation, and sick leave during the year prior to COVID-19 infection. The significance level was set to $p < 0.05$.

RESULTS

Study sample

Out of 11,955 individuals in the cohort, 5464 individuals completed the survey, corresponding to a response rate of 45.7%, 4240 of whom completed the survey via the web. The mean age of the responders was 51 years, 66% of whom were women. Among responders, the mean number of days on sick leave during the first year following COVID-19 infection was 67 (SD 91.8), and approximately one in four had been hospitalised due to COVID-19 (Table 1).

Responders were older, had longer duration of sick leave due to COVID-19, were more likely to have been hospitalised due to COVID-19, were more often women, and had higher education and income levels than non-responders. There was no significant difference in sick leave prior to COVID-19 between responders and non-responders (Table 1).

For peer review only - http://bmjopen.bmj.com/site/about/guidelines.xhtml

For peer review only

TABLE 1 Characteristics of responders and non-responders.

	**Responders** *n* = 5464 (45.7%)	**Non-responders** *n* = 6491 (54.3%)	***p*-value**
**Age**, mean (SD), years	50.85 (10.21)	45.60 (11.62)	<0.001
**Sex**, *n* (%)			
Men	1865 (34.1)	2961 (45.6)	
Women	3599 (65.9)	3530 (54.4)	<0.001
**Education level**, *n* (%)			
Primary school (≤9 years)	347 (6.4)	890 (13.9)	
Secondary school (10–12 years)	2598 (47.6)	3291 (51.3)	
Short university education (13–14 years)	841 (15.4)	902 (14.1)	<0.001
Long university education (≥15 years)	1667 (30.6)	1328 (20.7)	
**Employment status**, *n* (%)			
Employed	5384 (98.5)	6306 (97.1)	
Unemployed	80 (1.5)	185 (2.9)	<0.001
**Income**, *n* (%)			
Low income	1428 (26.1)	2555 (39.4)	
Medium income	1900 (34.8)	2085 (32.1)	
High income	2136 (39.1)	1849 (28.5)	<0.001
**Hospitalisation due to COVID-19**, *n* (%)	1503 (27.5)	1457 (22.4)	<0.001
**Days of sick leave**			
Mean (SD)	67.28 (91.80)	57.80 (74.55)	<0.001
Median (IQR)	36 (28)	35 (24)	0.003
**Sick leave prior to COVID-19**, *n* (%)	856 (15.7)	1076 (16.6)	0.178

Abbreviations: SD, standard deviation; IQR, interquartile range

Physical activity

PA level was lower in hospitalised responders than in non-hospitalised responder both prior to COVID-19 (*p* = 0.035) and at the 18-month follow-up (*p* = 0.008; Table 2).

TABLE 2. Current and previous physical activity levels in the responders.

	**Responders**	**Responders hospitalised due to COVID-19**	**Responders non-hospitalised due to COVID-19**	***p*-value***
**Physical activity level prior to COVID-19 infection**	5175 (94.7)	1438 (95.6)	3737 (94.3)	
Vigorous physical activity	455 (8.8)	111 (7.7)	344 (9.2)	
Moderate physical activity	2085 (40.3)	560 (38.9)	1525 (40.8)	
Light physical activity	2252 (43.5)	642 (44.6)	1610 (43.1)	0.035
Physically inactive	383 (7.4)	125 (8.7)	258 (6.9)	
**Current physical activity level**	5187 (94.9)	1437 (95.6)	3750 (94.7)	
Vigorous physical activity	159 (3.1)	39 (2.7)	120 (3.2)	
Moderate physical activity	1369 (26.4)	395 (27.5)	974 (26.0)	0.008
Light physical activity	2782 (53.6)	726 (50.5)	2056 (54.8)	

For peer review only - http://bmjopen.bmj.com/site/about/guidelines.xhtml

Physically inactive	877 (16.9)	277 (19.3)	600 (16.0)	

Values are given as n (%). * Mann Whitney-U test

In the whole group of responders, the PA level decreased from prior COVID-19 infection to the 18-month follow-up (p < 0.001; Figure 1). Summing up the changes in PA, the level of PA decreased in 1726 (33.6%) responders, was sustained in 3105 (60.5%) responders, and increased in 300 (5.8%) responders. Of the individuals with the highest level of PA (SGPALS 4) prior to COVID-19, approximately one-third sustained their level of PA until the 18-month follow-up. In the subgroup analysis based on acute disease severity, the PA level decreased in the non-hospitalised group (p < 0.001), with 1244 responders (33.5%) decreasing their level of PA, 2282 (61.5%) sustaining their level of PA, and 184 (5.1%) increasing their level of PA. A significant decrease in PA was also found in the initially hospitalised responders (p < 0.001), with 482 (33.9%) decreasing their level of PA, 823 (58.0%) sustaining their level of PA, and 115 (8.1%) increasing their level of PA. There was no difference in change in PA over time between hospitalised and non-hospitalised responders (p=0.193).

Insert figure 1 about here

Long-term consequences of COVID-19 in daily life and relationship to PA

At the 18-month follow-up, 2434 responders (46%) reported still having difficulties performing daily life activities due to COVID-19. Logistic regression was performed to explain these long-term difficulties. The model showed that a decrease in PA increased the odds (OR 5.58, 95% CI 4.90–6.34) of having difficulties performing daily life activities. Furthermore, having been initially hospitalised due to COVID-19 and taking sick leave during the year prior to COVID-19 also increased the odds, whereas age and sex did not contribute significantly to long-term difficulties in daily life activities (Figure 2). The model could significantly (p <0.001) distinguish between responders who did or did not report perceived difficulties in performing daily life activities at the 18-month follow-up.

Insert figure 2 about here

DISCUSSION

The results of this study show that individuals with less severe acute disease had a higher level of PA, both prior to COVID-19 and at the 18-month follow-up, than individuals with more severe acute

For peer review only - http://bmjopen.bmj.com/site/about/guidelines.xhtml

disease. In both the total study population and both groups based on acute disease severity, the PA level was decreased at the 18-month follow-up compared with the level prior to COVID-19. Furthermore, a decrease in PA over that time was associated with perceived difficulties performing daily life activities 18 months after COVID-19.

The PA level of responders was rather high before COVID-19. The study population also seems to be quite physically active compared with other cohorts in which PA has been assessed by the same measure (24-26). Given that regular PA has been shown to be beneficial in protecting individuals against severe illness and hospitalisation for COVID-19 (11, 12), we could anticipate that this population would not be severely affected by COVID-19 infection. Nevertheless, one-fourth of the current study population was initially hospitalised. Being more physically active during the pandemic may have entailed being in places or contexts with a higher risk of transmission of COVID-19, such as gyms or team sports, whereas less active individuals may have been more isolated in their own homes. Furthermore, exercising while already infected with COVID-19 may have worsened the course of the disease (27). In the polio epidemics, being very physically active (28) or pregnant (29), both of which affect the immune system, increased the risk of paralysis among poliomyelitis cases. However, in the current study population's subgroups based on acute disease severity, hospitalised responders had significantly lower PA levels than non-hospitalised responders, supporting previous findings of the benefits of PA in regard to COVID-19 severity (11, 12).

Summing up the changes in PA, there was a decrease in the level of PA in both the total study population and the groups based on acute disease severity. In the general Swedish population, PA increased during the pandemic (13-15), in contrast to the current findings in a cohort of individuals who had COVID-19. The decrease in PA seen in the current study can be speculated to relate to the disease itself and deconditioning (30). However, a Swedish study (14) reported that, in a general population, 36% reported increased PA and 30% decreased PA during the pandemic; 71% and 89%, respectively, attributed this change to COVID-19–related restrictions (14). The relatively mild approach to restrictions in Sweden may have had diverse consequences for PA in different groups of individuals. Recommendations, such as working from home, may have given them more free time and, therefore, increased PA for some, whereas cancelled sporting events and competitions may have had the opposite effect, especially among individuals with a high level of PA. In the current study, only one-third of individuals with the highest level of PA prior to COVID-19 sustained their PA level until the 18-month follow-up.

Furthermore, the strictest recommendations, which involved isolation and were applied to people >70 years of age and those infected, as a means to protect the most vulnerable and constrain

For peer review only - http://bmjopen.bmj.com/site/about/guidelines.xhtml

transmission, most likely had an influence on the levels of PA. A study from the US showed an association between self-isolation and negative mental health that was not seen with social distancing (18). Previous studies (13, 17, 18) also reported an association between negative mental health effects and a decrease in PA during the pandemic. Taken together, these findings suggest that pandemic-related restrictions and mental health could also play a role in the decrease in PA in the current cohort.

In this study, in-hospital care due to COVID-19 was used as a proxy for acute disease severity. As no one in Sweden was denied in-hospital care due to lack of capacity and healthcare in Sweden is tax-funded, this proxy for acute disease severity seems reasonable and relies on the assessment of educated healthcare professionals. Furthermore, need for hospital care is included in the WHO Clinical Progression Scale (31), which supports the interpretation of in-hospital care as a proxy for COVID-19 disease severity.

Using the SGPALS (23), the current study showed a decrease in PA among individuals who were on sick leave due to COVID-19 during the first wave of the pandemic in Sweden. Notably, only one-third of individuals with the highest level of PA prior to COVID-19 sustained their PA level until the 18-month follow-up. Because making up for deconditioning takes time (32), this decline in PA may have long-term implications, and returning to pre–COVID-19 activities and levels may be difficult even for the most highly trained individuals, such as elite athletes (33).

PA is important for maintaining health and can protect against both non-communicable chronic diseases (34) and communicable diseases (35), such as COVID-19. Therefore, the results of this study, with a decreased PA level regardless of acute disease severity, are worrisome, especially considering the risk of future pandemics.

A strength of the present study was that a validated instrument, the SGPALS, was used for questions regarding PA. Furthermore, the different steps of the scale were clearly defined in the questionnaire, making it easier for individuals to answer according to their true activity levels. This lends weight to the results and facilitates comparisons with other studies and populations. However, the retrospective assessment of PA level, asking responders to report their PA level 18 months previous, involves risk or recall bias, which is a limitation.

Another limitation of the study was the response rate of nearly 50%, and responders were found to be older and having had more severe acute disease than non-responders. Thus, differences between responders and non-responders need to be considered when interpreting the findings.

For peer review only - http://bmjopen.bmj.com/site/about/guidelines.xhtml

In conclusion, PA levels were reduced 18 months after COVID-19 infection. A decrease in PA over that time was associated with perceived difficulties performing daily life activities 18 months after COVID-19. As PA is important in maintaining health and deconditioning takes time to reverse, this decline may have long-term implications for PA and health.

Author contributions

AP contributed to the conception and design of the study, managed data acquisition and analysis, contributed to interpreting the data and drafted the work.
JS contributed to interpreting and presenting the data, and drafting the work.
HP contributed to the conception, design, and acquisition of the study, as well as data management and interpretation.
KSS contributed to the conception, design, and acquisition of the study, as well as interpretation of the data.
All authors reviewed the work critically for important intellectual content and approved the final version to be published.

For peer review only

For peer review only - http://bmjopen.bmj.com/site/about/guidelines.xhtml

Figure legends:

FIGURE 1 Changes in physical activity (PA) levels according to the Saltin–Grimby PA Level Scale (SGPALS) from prior to COVID-19 infection (left side) to the 18-month follow-up (right side) *n* = 5131 individuals. SGPALS: 1, physically inactive; 2, light PA for at least 4 hours/week; 3, regular moderate PA and training for at least 2–3 hours/week; and 4, regular vigorous physical training for competitive sports several times per week.

FIGURE 2. Variables in the model explaining the odds of perceiving difficulties in performing daily life activities. The model explained between 15% (Cox & Snell R^2) and 20% (Nagelkerke R^2) of the variance in the outcome.

For peer review only

For peer review only - http://bmjopen.bmj.com/site/about/guidelines.xhtml

References

1. Ai X, Yang J, Lin Z, et al. Mental Health and the Role of Physical Activity During the COVID-19 Pandemic. Front Psychol. 2021;12:759987.10.3389/fpsyg.2021.759987.
2. Ravalli S, Musumeci G. Coronavirus Outbreak in Italy: Physiological Benefits of Home-Based Exercise During Pandemic. J Funct Morphol Kinesiol. 2020;5(2).10.3390/jfmk5020031.
3. Gupta A, Madhavan MV, Sehgal K, et al. Extrapulmonary manifestations of COVID-19. Nat Med. 2020;26(7):1017-32.10.1038/s41591-020-0968-3.
4. Wiersinga WJ, Rhodes A, Cheng AC, et al. Pathophysiology, Transmission, Diagnosis, and Treatment of Coronavirus Disease 2019 (COVID-19): A Review. Jama. 2020;324(8):782-93.10.1001/jama.2020.12839.
5. Higgins V, Sohaei D, Diamandis EP, et al. COVID-19: from an acute to chronic disease? Potential long-term health consequences. Crit Rev Clin Lab Sci. 2021;58(5):297-310.10.1080/10408363.2020.1860895.
6. Ballering AV, van Zon SKR, Olde Hartman TC, et al. Persistence of somatic symptoms after COVID-19 in the Netherlands: an observational cohort study. Lancet. 2022;400(10350):452-61.10.1016/s0140-6736(22)01214-4.
7. O'Mahoney LL, Routen A, Gillies C, et al. The prevalence and long-term health effects of Long Covid among hospitalised and non-hospitalised populations: A systematic review and meta-analysis. EClinicalMedicine. 2023;55:101762.10.1016/j.eclinm.2022.101762.
8. Wahlgren C, Forsberg G, Divanoglou A, et al. Two-year follow-up of patients with post-COVID-19 condition in Sweden: a prospective cohort study. Lancet Reg Health Eur. 2023;28:100595.10.1016/j.lanepe.2023.100595.
9. World Health Organization. WHO guidelines on physical activity and sedentary behaviour 2020.
10. da Silveira MP, da Silva Fagundes KK, Bizuti MR, et al. Physical exercise as a tool to help the immune system against COVID-19: an integrative review of the current literature. Clin Exp Med. 2021;21(1):15-28.10.1007/s10238-020-00650-3.
11. Ezzatvar Y, Ramírez-Vélez R, Izquierdo M, et al. Physical activity and risk of infection, severity and mortality of COVID-19: a systematic review and non-linear dose-response meta-analysis of data from 1 853 610 adults. Br J Sports Med. 2022.10.1136/bjsports-2022-105733.
12. Sallis R, Young DR, Tartof SY, et al. Physical inactivity is associated with a higher risk for severe COVID-19 outcomes: a study in 48 440 adult patients. Br J Sports Med. 2021;55(19):1099-105.10.1136/bjsports-2021-104080.
13. Blom V, Lönn A, Ekblom B, et al. Lifestyle Habits and Mental Health in Light of the Two COVID-19 Pandemic Waves in Sweden, 2020. Int J Environ Res Public Health. 2021;18(6).10.3390/ijerph18063313.
14. Eek F, Larsson C, Wisén A, et al. Self-Perceived Changes in Physical Activity and the Relation to Life Satisfaction and Rated Physical Capacity in Swedish Adults during the COVID-19 Pandemic-A Cross Sectional Study. Int J Environ Res Public Health. 2021;18(2).10.3390/ijerph18020671.
15. Brattlöf F, Gustafsson PE, San Sebastián M. Prevalence and change in social inequalities in physical activity before and during the COVID-19 pandemic in Sweden. Int J Equity Health. 2023;22(1):21.10.1186/s12939-023-01835-4.
16. McCarthy H, Potts HWW, Fisher A. Physical Activity Behavior Before, During, and After COVID-19 Restrictions: Longitudinal Smartphone-Tracking Study of Adults in the United Kingdom. J Med Internet Res. 2021;23(2):e23701.10.2196/23701.
17. Maugeri G, Castrogiovanni P, Battaglia G, et al. The impact of physical activity on psychological health during Covid-19 pandemic in Italy. Heliyon. 2020;6(6).
18. Meyer J, McDowell C, Lansing J, et al. Changes in Physical Activity and Sedentary Behavior in Response to COVID-19 and Their Associations with Mental Health in 3052 US Adults. Int J Environ Res Public Health. 2020;17(18).10.3390/ijerph17186469.

For peer review only - http://bmjopen.bmj.com/site/about/guidelines.xhtml

19. Calvo-Bonacho E, Catalina-Romero C, Fernández-Labandera C, et al. COVID-19 and Sick Leave: An Analysis of the Ibermutua Cohort of Over 1,651,305 Spanish Workers in the First Trimester of 2020. Front Public Health. 2020;8:580546.10.3389/fpubh.2020.580546.
20. Westerlind E, Palstam A, Sunnerhagen KS, et al. Patterns and predictors of sick leave after Covid-19 and long Covid in a national Swedish cohort. BMC Public Health. 2021;21(1):1023.10.1186/s12889-021-11013-2.
21. Palstam A, Westerlind E, Sunnerhagen KS, et al. Recurrent sick leave after COVID-19: investigating the first wave of the pandemic in a comprehensive Swedish registry-based study. BMC Public Health. 2021;21(1):1914.10.1186/s12889-021-11918-y.
22. Saltin B, Grimby G. Physiological analysis of middle-aged and old former athletes: comparison with still active athletes of the same ages. Circulation. 1968;38(6):1104-15.
23. Grimby G, Börjesson M, Jonsdottir I, et al. The "Saltin–Grimby physical activity level scale" and its application to health research. Scand J Med Sci Sports. 2015;25:119-25.
24. Mikkilä S, Johansson J, Nordström A, et al. A 15-year follow-up study of hip bone mineral density and associations with leisure time physical activity. The Tromsø Study 2001-2016. PLoS One. 2022;17(1):e0262228.10.1371/journal.pone.0262228.
25. Rödjer L, I HJ, Börjesson M. Physical activity on prescription (PAP): self-reported physical activity and quality of life in a Swedish primary care population, 2-year follow-up. Scand J Prim Health Care. 2016;34(4):443-52.10.1080/02813432.2016.1253820.
26. Gustavsson J, Mehlig K, Leander K, et al. FTO genotype, physical activity, and coronary heart disease risk in Swedish men and women. Circ Cardiovasc Genet. 2014;7(2):171-7.10.1161/circgenetics.111.000007.
27. Nieman DC. Coronavirus disease-2019: A tocsin to our aging, unfit, corpulent, and immunodeficient society. Journal of sport and health science. 2020;9(4):293-301.
28. Friman G, Wesslén L. Infections and exercise in high-performance athletes. Immunology and cell biology. 2000;78(5):510-22.
29. Siegel M, Greenberg M. Incidence of poliomyelitis in pregnancy: its relation to maternal age, parity and gestational period. New England Journal of Medicine. 1955;253(20):841-7.
30. Rinaldo RF, Mondoni M, Parazzini EM, et al. Deconditioning as main mechanism of impaired exercise response in COVID-19 survivors. Eur Respir J. 2021;58(2).10.1183/13993003.00870-2021.
31. WHO Working Group on the Clinical Characterisation and Management of COVID-19 infection. A minimal common outcome measure set for COVID-19 clinical research. Lancet Infect Dis. 2020;20(8):e192-e7.10.1016/s1473-3099(20)30483-7.
32. Blomquist G. Respons to exercise after bed rest and training. Circulation. 1968;38(7).
33. Williams Z, Hull JH. Respiratory complications following COVID-19 in athletic populations: A narrative review. Scandinavian Journal of Medicine & Science in Sports. 2022.
34. Lee IM, Shiroma EJ, Lobelo F, et al. Effect of physical inactivity on major non-communicable diseases worldwide: an analysis of burden of disease and life expectancy. Lancet. 2012;380(9838):219-29.10.1016/s0140-6736(12)61031-9.
35. Chastin SFM, Abaraogu U, Bourgois JG, et al. Effects of Regular Physical Activity on the Immune System, Vaccination and Risk of Community-Acquired Infectious Disease in the General Population: Systematic Review and Meta-Analysis. Sports Med. 2021;51(8):1673-86.10.1007/s40279-021-01466-1.

For peer review only - http://bmjopen.bmj.com/site/about/guidelines.xhtml

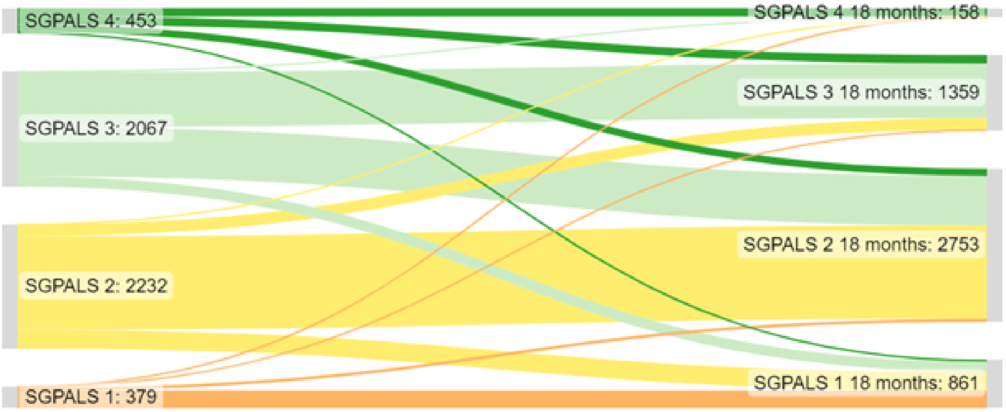

FIGURE 1 Changes in physical activity (PA) levels according to the Saltin–Grimby PA Level Scale (SGPALS) from prior to COVID-19 infection (left side) to the 18-month follow-up (right side) n = 5131 individuals. SGPALS: 1, physically inactive; 2, light PA for at least 4 hours/week; 3, regular moderate PA and training for at least 2–3 hours/week; and 4, regular vigorous physical training for competitive sports several times per week.

404x187mm (38 x 38 DPI)

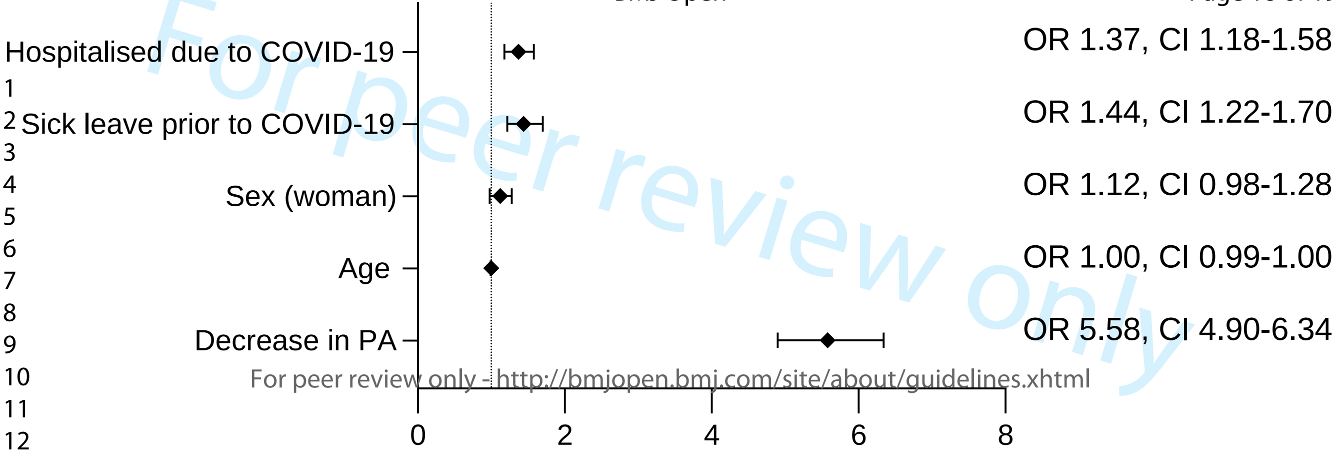

For peer review only - http://bmjopen.bmj.com/site/about/guidelines.xhtml

STROBE 2007 (v4) checklist of items to be included in reports of observational studies in epidemiology*

Checklist for cohort, case-control, and cross-sectional studies (combined)

Section/Topic	Item #	Recommendation	Reported on page #
**Title and abstract**	1	(*a*) Indicate the study's design with a commonly used term in the title or the abstract	1
		(*b*) Provide in the abstract an informative and balanced summary of what was done and what was found	2
**Introduction**			
Background/rationale	2	Explain the scientific background and rationale for the investigation being reported	3-4
Objectives	3	State specific objectives, including any pre-specified hypotheses	4
**Methods**			
Study design	4	Present key elements of study design early in the paper	4
Setting	5	Describe the setting, locations, and relevant dates, including periods of recruitment, exposure, follow-up, and data collection	4-5
Participants	6	(*a*) *Cohort study*—Give the eligibility criteria, and the sources and methods of selection of participants. Describe methods of follow-up<br>*Case-control study*—Give the eligibility criteria, and the sources and methods of case ascertainment and control selection. Give the rationale for the choice of cases and controls<br>*Cross-sectional study*—Give the eligibility criteria, and the sources and methods of selection of participants	4-5
		(*b*) *Cohort study*—For matched studies, give matching criteria and number of exposed and unexposed<br>*Case-control study*—For matched studies, give matching criteria and the number of controls per case	
Variables	7	Clearly define all outcomes, exposures, predictors, potential confounders, and effect modifiers. Give diagnostic criteria, if applicable	4-5
Data sources/ measurement	8*	For each variable of interest, give sources of data and details of methods of assessment (measurement). Describe comparability of assessment methods if there is more than one group	4-5
Bias	9	Describe any efforts to address potential sources of bias	6
Study size	10	Explain how the study size was arrived at	5
Quantitative variables	11	Explain how quantitative variables were handled in the analyses. If applicable, describe which groupings were chosen and why	6
Statistical methods	12	(*a*) Describe all statistical methods, including those used to control for confounding	6
		(*b*) Describe any methods used to examine subgroups and interactions	6
		(*c*) Explain how missing data were addressed	6
		(*d*) *Cohort study*—If applicable, explain how loss to follow-up was addressed<br>*Case-control study*—If applicable, explain how matching of cases and controls was addressed	6

For peer review only

		Cross-sectional study—If applicable, describe analytical methods taking account of sampling strategy	
		(*e*) Describe any sensitivity analyses	
**Results**			
Participants	13*	(a) Report numbers of individuals at each stage of study—eg numbers potentially eligible, examined for eligibility, confirmed eligible, included in the study, completing follow-up, and analysed	6
		(b) Give reasons for non-participation at each stage	6
		(c) Consider use of a flow diagram	
Descriptive data	14*	(a) Give characteristics of study participants (eg demographic, clinical, social) and information on exposures and potential confounders	6
		(b) Indicate number of participants with missing data for each variable of interest	6-8
		(c) *Cohort study*—Summarise follow-up time (eg, average and total amount)	6
Outcome data	15*	*Cohort study*—Report numbers of outcome events or summary measures over time	
		Case-control study—Report numbers in each exposure category, or summary measures of exposure	
		Cross-sectional study—Report numbers of outcome events or summary measures	6-8
Main results	16	(*a*) Give unadjusted estimates and, if applicable, confounder-adjusted estimates and their precision (eg, 95% confidence interval). Make clear which confounders were adjusted for and why they were included	8
		(*b*) Report category boundaries when continuous variables were categorized	
		(*c*) If relevant, consider translating estimates of relative risk into absolute risk for a meaningful time period	
Other analyses	17	Report other analyses done—eg analyses of subgroups and interactions, and sensitivity analyses	
**Discussion**			
Key results	18	Summarise key results with reference to study objectives	8
Limitations	19	Discuss limitations of the study, taking into account sources of potential bias or imprecision. Discuss both direction and magnitude of any potential bias	3, 9
Interpretation	20	Give a cautious overall interpretation of results considering objectives, limitations, multiplicity of analyses, results from similar studies, and other relevant evidence	10
Generalisability	21	Discuss the generalisability (external validity) of the study results	3, 9-10
**Other information**			
Funding	22	Give the source of funding and the role of the funders for the present study and, if applicable, for the original study on which the present article is based	1

*Give information separately for cases and controls in case-control studies and, if applicable, for exposed and unexposed groups in cohort and cross-sectional studies.

Note: An Explanation and Elaboration article discusses each checklist item and gives methodological background and published examples of transparent reporting. The STROBE checklist is best used in conjunction with this article (freely available on the Web sites of PLoS Medicine at http://www.plosmedicine.org/, Annals of Internal Medicine at http://www.annals.org/, and Epidemiology at http://www.epidem.com/). Information on the STROBE Initiative is available at www.strobe-statement.org.

For peer review only
